# How healthcare providers and the right information may play a critical role in quitting success among smokers interested in using e-cigarettes for quitting: Results from a survey of U.S adults

**DOI:** 10.1371/journal.pone.0303245

**Published:** 2024-05-16

**Authors:** Akshika Sharma, Jaelen King, Suchitra Krishnan-Sarin, Stephanie S. O’Malley, Meghan Morean, Krysten Bold

**Affiliations:** Department of Psychiatry, Yale School of Medicine, Yale University, New Haven, CT, United States of America; University of Pennsylvania, UNITED STATES

## Abstract

**Introduction:**

Promoting smoking cessation is a global public health priority. E-cigarettes are increasingly being used by individuals to try quitting smoking. Identifying sources and types of information available to adults who are trying to quit, and the impact of this information during a quit attempt, is critical to augment the potential public health benefit of e-cigarettes for reducing cigarette smoking.

**Methods:**

US adults (N = 857) who reported using e-cigarettes in a recent smoking cessation attempt completed an anonymous, cross sectional, online survey. We examined sources of information and type of information received when using e-cigarettes to quit smoking and their associations with the duration of abstinence achieved.

**Results:**

The two most commonly reported information sources were friends (43.9%) and the internet (35.2%), while 14.0% received information from a healthcare provider. People received information on type of device (48.5%), flavor (46.3%), and nicotine concentration (43.6%). More people received information about gradually switching from smoking to vaping (46.7%) than abruptly switching (30.2%). Obtaining information from healthcare providers (β (SE) = 0.16 (0.08), *p* = 0.04), getting information about abruptly switching to e-cigarettes (β (SE) = 0.14 (0.06), *p* = 0.01) and what nicotine concentrations to use (β (SE) = 0.18 (0.05), *p* = 0.03) were associated with longer quit durations.

**Conclusions:**

Amidst the growing popularity of e-cigarettes use for quitting smoking, our results highlight common sources of information and types of information received by individuals. Few people received information from healthcare providers indicating a gap in cessation support that can be filled. Providing information about immediate switching to e-cigarettes and nicotine concentrations to use may help in increasing quit rates and duration.

## Introduction

Cigarette smoking continues to be a health risk globally and within in the United States, with more than 4,80,000 Americans dying each year from smoking-related conditions [1]. A large percentage of people who smoke cigarettes express interest in quitting, but they struggle to achieve and sustain their goal [2, 3]. There are numerous cessation options available that have led to varying levels of success in quitting [4]. Although not FDA-approved, one relatively new method that has shown promise is the use of e-cigarettes [5, 6].

E-cigarettes were initially introduced and marketed as a reduced-harm alternative for adults who smoke cigarettes. Studies suggest that many adults who smoke cigarettes report e-cigarette use as a way to try to quit smoking. Accumulating studies provide evidence that e-cigarette use is associated with more quit attempts and greater quit success for adults who smoke compared to nicotine replacement therapy (NRT) or behavioral therapies only [6–11]. Although many people try e-cigarettes for quitting smoking, not all succeed, and some become dual users which may not reduce harm. Thus, we need a better understanding of the factors associated with smoking outcomes when using e-cigarettes to quit, such as the sources of information and types of information people rely on in doing so. There is limited knowledge about where people receive information about using e-cigarettes to quit smoking and what types of information they receive [12, 13]. Identifying e-cigarette-related information that might be relevant for adults during a cessation attempt and where they receive that information may be important for maximizing the potential for e-cigarettes to reduce smoking-related harm. Prior research has reported common information sources for awareness and general knowledge of e-cigarettes (e.g., from others who use e-cigarettes, media advertisements, the news) [13]. However, little is known about sources that are used specifically to obtain information about using e-cigarettes as smoking cessation aids and the type of information that individuals receive from them. Understanding more about how people decide to use e-cigarettes to quit smoking and specific information about using e-cigarettes that is found to be useful could provide important guidance to support smoking cessation attempts and improve public health. This paper aims to highlight the importance of information sources and the types of information received during quit attempts using e-cigarettes and their relations to quit duration.

## Methods

### Participants and procedures

A web-based survey of US adults was conducted from May to August 2021. A detailed account of the procedures is explained elsewhere [14]. Briefly, Qualtrics survey panels were used to recruit adults who smoked cigarettes and reported using e-cigarettes in a recent attempt to quit smoking. Eligibility criteria included living in the U.S., being at least 21 years old, reporting a history of regular cigarette smoking (defined as smoking ≥ 1 year, ≥ 4 days/week), and using e-cigarettes in a quit smoking attempt within the past 2 years. Participants were compensated directly by Qualtrics. All procedures were approved by the Yale University Institutional Review Board (IRB). The study was determined to be exempt from continued IRB review since the data collected did not include identifying information.

### Measures

#### Demographics

Participants reported on age, biological sex, state of residence (which was mapped onto the census quadrants of West, Midwest, Northeast, and South), ethnicity, race, level of educational attainment, and subjective financial situation as an index of socioeconomic status (SES) [15].

#### Sources of information about how to use e-cigarettes to quit smoking

Participants were asked ‘Where did you get advice or information about how to use e-cigarettes/vapes to quit smoking?’. Participants could select all that applied from the following options: a vape shop employee, a friend, a coworker/colleague, a family member, a healthcare provider, an internet search, social media, other (please describe), and ‘I did not get any specific information on how to use e-cigarettes/vapes to quit smoking’.

#### Type(s) of information received on how to use e-cigarettes to quit smoking

Participants who endorsed receiving information from any source in the above question (n = 777) (i.e., who did not respond ‘I did not get any specific information on how to use e-cigarettes/vapes to quit smoking’) were asked, ‘What type of advice or information did you receive about how to use e-cigarettes/vapes to quit smoking?’. Participants could select all that applied from the following options: What kind of device to use (e.g., a JUUL, a mod, a vape-pen), What flavor or flavors to use, What nicotine concentration/strength to use, Making an abrupt switch from smoking to vaping (e.g., quit smoking and start vaping on the same day), Slowly switch from smoking to vaping (e.g., cutting back on smoking while slowly increasing your vaping), Other (please describe), and ‘I did not get any specific guidance on how to use e-cigarettes/vapes to quit smoking.’

#### Quit duration

Participants were asked ‘In the past 2 years when you were using an e-cigarette/vape to try and quit smoking, what is the longest amount of time you went without smoking a cigarette?’ Responses were obtained as categories from less than 1 day to more than 1 year (less than a day, less than 1 week, 1–3 weeks, 1 month, 2–3 months, 4–6 months, 7–9 months, 10–12 months, and More than a year), and responses were coded into a single continuous measure [14] that was log transformed for analysis.

### Data analysis

Frequency distributions were calculated for responses for all study variables. Two linear regression models were used with duration of abstinence achieved as the outcome. The first model included all sources of information as predictors, and the second model included all types of information received as the predictors. Both models were adjusted for race, SES, geographic location, and cigarette dependence. In our data, SES showed a moderate positive correlation with education, and based on previously reported literature, we decided to use SES as a proxy for education [16–18]. When we include age and sex in the models, our primary predictors (obtaining information from a healthcare provider or obtaining information about abrupt switching) were no longer significantly associated with quit duration. In our initial analysis exploring this, we observed that younger age and male sex were highly correlated with these predictors (i.e. obtaining information from a healthcare provider as well as with getting information about abrupt switching from cigarettes to e-cigarettes), so we did not include these demographics in the final regression models.

The participants could select multiple response options for both sources and types of information. Therefore, source of information and type of information were examined separately, and it was not feasible to examine the impact of source of information by type of information received due to statistical limitations in cases of small sample sizes selecting exclusively one source of information. Analyses were performed using SPSS (version 28.0).

## Results

### Demographics

A total of 857 participants completed the survey [mean (SD) = 40.8 (±12.3)] years; 52% male; cigarettes smoked per day [11.6 (±7.6)]; average duration of smoking in years [(17.5 (±12.7)]. Based on small sample sizes within certain ethnic/racial groups, we categorized participants as non-Hispanic White (62.8%), Hispanic (21.7%), non-Hispanic Black (7.7%), and non-Hispanic “other” (7.8%), which comprised Native Americans, Pacific Islander, Asian, and Multiracial groups.

### Sources of advice about using e-cigarettes to quit smoking

A friend was the most common source of advice (43.9%), followed by the internet (35.2%), social media (32.7%), a vape shop employee (28.1%), a family member (26.4%), a coworker/colleague (19.3%), and a healthcare provider (14.0%). 9.3% of participants indicated they did not get any information (Table 1). When all sources of information were entered into an adjusted linear regression model predicting quit duration, receiving information about using e-cigarettes to quit smoking from a healthcare provider uniquely was associated with longer a quit duration (β (SE) = 0.16 (0.08), *p* = 0.04). This association remains significant in an unadjusted model (Table 2).

**Table 1:** Sources of information accessed, and types of information received about using e-cigarette for quitting smoking.

Sources of information about e-cigarettes for quitting	Overall Response Count^1^ (N = 857) N (%)
A friend	376 (43.9)
Internet search	302 (35.2)
Social media	280 (32.7)
A vape shop employee	241 (28.1)
A family member	226 (26.4)
A coworker/ colleague	165 (19.3)
A health care provider	120 (14.0)
Other^2^	6 (0.7)
Did not get any specific information	80 (9.3)
**Types of information received for e-cigarettes for quitting**	**Overall Response Count (n = 777)** **n (%)**
What kind of device to use	377 (48.5)
What flavors to use	360 (46.3)
What nicotine concentrations or strengths to use	339 (43.6)
Slowly switching from smoking to vaping	363 (46.7)
Making abrupt switch from smoking to vaping	235 (30.2)
Other	2 (0.2)
Did not get any specific guidance on how to use e-cigarettes	49 (6.3)

Footnotes: 1. People could select all that apply from sources of information, so values may add up to more than 100%. 2. Other responses included write ins: “myself,”, “my boyfriend’,” “coupon in mail to try for free,” “pamphlet that came with the product,” and “fuel station.”

**Table 2:** Regression model showing associations of information sources as predictors with outcome of quit duration.

Sources of information	Unadjusted values			Adjusted values^1^		
	B (S.E)	95% CI		B (S.E)	95% CI	
		Lower	Upper		Lower	Upper
A vape shop employee	0.07 (0.06)	-0.04	0.18	0.08 (0.06)	-0.04	0.19
A friend	-0.02 (0.05)	-0.12	0.08	-0.01 (0.05)	-0.11	0.09
A coworker/ colleague	-0.05 (0.07)	-0.17	0.08	-0.05 (0.07)	-0.18	0.08
A family member	-0.03 (0.06)	-0.14	0.08	-0.01 (0.06)	-0.13	0.10
A healthcare provider	0.16 (0.08) *	0.01	0.31	0.16 (0.08) *	0.01	0.30
An internet search	-0.02 (0.06)	-0.13	0.09	-0.05 (0.06)	-0.16	0.06
Social media	0.06 (0.06)	-0.05	0.17	0.05 (0.06)	-0.06	0.16

**^1^**Adjusted for covariates race, geographic location, SES, and cigarette dependence. * Significant at p<0.05.

### Type of information received on using e-cigarettes to quit smoking

Participants reported getting information about using specific e-cigarette devices (48.5%), flavors (46.3%), and nicotine concentrations (43.6%). More participants reported getting information about slowly switching from smoking to vaping (46.7%) than abruptly switching from cigarettes to e-cigarettes (30.2%). 6.3% participants reported not getting any specific information (Table 1). When all types of information received were entered into an adjusted linear regression model predicting quit duration, receiving information about abrupt switching to e-cigarettes for quitting smoking was associated with longer reported quit durations (β (SE) = 0.14 (0.06), *p* = 0.01), while receiving information about gradually switching from cigarettes to e-cigarettes was associated with shorter quit durations (β (SE) = -0.12 (0.05), *p* = 0.02). Additionally, receiving information about nicotine concentrations and strengths to use was associated with a longer quit duration (β (SE) = 0.18 (0.05), *p* = 0.03). In an unadjusted liner regression model, the association of receiving information about abrupt switching with longer quit duration and receiving information about gradual switching with shorter quit duration remain significant (Table 3). We added a supplemental table [1] on the type of information for using e-cigarettes for quitting by source for those individuals who reported receiving information exclusively from one source. However, the sample sizes were small for many information sources.

**Table 3:** Regression model showing associations of types of information received as predictors with outcome of quit duration.

Types of information received	Unadjusted values			Adjusted values^1^		
	B (S.E)	95% CI		B (S.E)	95% CI	
		Lower	Upper		Lower	Upper
What kind of device to use	0.04 (0.05)	-0.07	0.14	0.08 (0.06)	-0.03	0.19
What flavors to use	0.04 (0.05)	-0.06	0.15	0.03 (0.05)	-0.08	0.13
What nicotine concentrations or strengths to use	0.09 (0.05)	-0.20	0.19	0.18 (0.05) *	0.01	0.22
Slowly switching from smoking to vaping	-0.13 (0.05) *	-0.24	-0.03	-0.12 (0.05) *	-0.23	-0.02
Making abrupt switch from smoking to vaping	0.13 (0.06) *	0.01	0.24	0.14 (0.06) *	0.03	0.26
Other	-0.79 (0.51)	-1.78	0.20	-0.74 (0.51)	-1.74	0.25
Did not get any specific guidance on how to use e-cigarettes	0.02 (0.12)	-0.22	0.25	0.08 (0.12)	-0.16	0.32

**^1^**Adjusted for covariates race, geographic location, SES, and cigarette dependence. * Significant at p<0.05.

## Discussion

The study aimed to assess common sources where people obtain information about using e-cigarettes to quit smoking and the type of information received among adults who used e-cigarettes in a recent smoking cessation attempt. Obtaining information from a credible source may be important for increasing quit success, as our findings indicate that those receiving advice from a healthcare provider reported longer quit duration. However, healthcare providers were the least reported information source. Given emerging evidence that e-cigarettes may be more effective at promoting cigarette cessation compared to traditional smoking cessation aids (e.g., nicotine replacement therapy) [6], research is needed to identify effective messaging that healthcare providers can share with their patients about using e-cigarettes for smoking cessation. Additionally, training health care providers to equip them with evidence-based knowledge about e-cigarette use and its potential role in smoking cessation may be helpful in this critical behavioral decision. Information provided by health care professionals may have a greater impact on adoption and appropriate use of e-cigarettes. The role of healthcare providers in increasing smoking cessation is well documented [19]. Additionally, our findings indicate having a primary care provider or access to healthcare providers may be instrumental in supporting quitting efforts by providing useful information. However, healthcare providers were also the least reported information source, and healthcare provider access is limited for some individuals. We did not assess ease of access to healthcare providers in our study and this would be an important area for future work.

The three sources of information endorsed most frequently in our study were a friend, the internet, and social media. These findings relate to existing literature showing that friends and family influence use of e-cigarettes, overall, [20] and the internet and social media are common sources for general information about e-cigarettes [21–23]. However, our findings also provide new information by showing that friends and online sources also are common places where people seek information specifically about using e-cigarettes to quit smoking. However, the accuracy of information disseminated through different channels should be assessed and increased. Obtaining information from a credible source may be important for increasing quit success as our findings indicate that those receiving advice from a healthcare provider reported longer quit duration. However, it is possible that those who received information from a health care provider may have been more motivated to take action to improve their health compared to those receiving information from more informal sources like friends or the internet. However, obtaining accurate information is essential and specific guidelines should be placed by public health officials using effective communication programs to ensure scientifically accurate information reaches consumers.

Additionally, our results identified common types of information received about how to use e-cigarettes to quit smoking. Participants most often received information related to e-cigarettes themselves, including specific devices, flavors, and nicotine concentrations to use. Participants also reported receiving information about the best approach to take when switching from smoking to vaping. Information about slowly switching to e-cigarettes from cigarettes was received more often than information recommending abruptly switching. However, receiving information about abrupt switching to e-cigarettes was associated with longer durations of abstinence achieved during the quit attempt. This latter finding is consistent with prior research suggesting that abrupt switching, either from cigarettes to e-cigarettes [14, 24] or from cigarettes to no nicotine products at all [25] is associated with better quitting outcomes. Additionally, receiving information about nicotine concentrations and strengths to use was observed to be associated with longer quit duration. However more information is needed to understand what specific nicotine concentrations or whether tailoring of nicotine concentrations to individuals’ use are associated with successful quitting. The results have implications for providing guidelines for using e-cigarettes when quitting to encourage complete and abrupt cigarette substitution, along with guidance for tailored nicotine concentrations to use to maximize the potential benefit of e-cigarettes for smoking cessation. As mentioned in our data analysis section, younger age and male sex showed a high correlation with obtaining information from health care providers and getting information about abrupt switching to e-cigarettes. Thus, these findings suggest that some individuals may be more likely to obtain information about using an e-cigarette to quit from a healthcare provider.

Findings should be considered in light of study limitations. Online surveys, by nature, have participants who self-select to enroll, which can limit the generalizability and external validity of results. Additionally, all responses were self-reported, with no biochemical verification. Participants’ responses were based on retrospective recall of their experiences, which may have introduced systematic error that biased the results. Thus, prospective longitudinal studies are required to assess the role of e-cigarettes in quitting smoking and the role of information sources and type of information provided to support smoking cessation efforts using e-cigarettes. We were unable to evaluate types of information provided by source because responses were ‘select all that apply’. Hence, further research is required to determine what types of information are provided by different sources and which strategies are most helpful for long-term quitting with use of e-cigarettes. A future focus on providing scientifically accurate information related to e-cigarettes as a smoking cessation tool may lead to increased attempts to use e-cigarettes to quit smoking, which could potentially lead to higher success rates [7, 26].

## Conclusion

Amidst the growing popularity of e-cigarettes use for quitting smoking, our results highlight common sources of information and types of information received by individuals. Few people received information from healthcare providers indicating a gap in cessation support that can be filled. However, receiving advice from a healthcare provider was also related to longer quit durations which may relate to quitting motivation, credibility of the source, or type of information provided. Additionally, people who received information about immediate switching to e-cigarettes and those receiving information about nicotine concentrations to use had longer quit durations, suggesting this may be an effective strategy to enhance quitting success and maximize the public health benefit of using e-cigarettes to quit smoking. This paper highlights critical findings which may help improve cessation and support services by providing valuable information about perceptions and use of e-cigarettes for quitting smoking among adults.

## Supporting information

S1 Dataset (XLSX)

S1 Table Type of information for using e-cigarettes for quitting by source for those individuals who reported receiving information exclusively from one source (n = 293). (DOCX)
