# Supplementary material for: How healthcare providers and the right information may play a critical role in quitting success among smokers interested in using e-cigarettes for quitting: Results from a survey of U.S adults
Source: PLoS One. 2024 May 16;19(5):e0303245. doi: 10.1371/journal.pone.0303245 (PMC11098412; doi:10.1371/journal.pone.0303245)
Supplement: S1 Table — (DOCX) [file pone.0303245.s002.docx]

| **Types of information** | **Sources of information** | | | | | | |
| --- | --- | --- | --- | --- | --- | --- | --- |
|  | Friend (n=98) | Internet search (n=52) | Social media (n=27) | Vape shop employee (n=42) | Family member (n=52) | Co-worker/ colleague (n=16) | Healthcare providers (n=6) |
| Device to use | 44 (44.9%) | 18 (34.6%) | 7 (25.9%) | 16 (38.1%) | 19 (36.5%) | 6  (37.5%) | 3  (0.5%) |
| Flavor | 29 (29.6%) | 22 (42.3%) | 8 (29.6%) | 15 (35.7%) | 18 (34.6%) | 3  (18.8%) | 1  (16.7%) |
| Nicotine concentration | 28 (28.6%) | 19 (36.5%) | 3 (11.1%) | 20 (47.6%) | 22 (42.3%) | 7  (43.8%) | 2  (33.3%) |
| Making an abrupt switch | 20 (20.4%) | 6 (11.5%) | 4 (14.8%) | 8  (19.0%) | 13 (25.0%) | 3  (18.8%) | 4  (66.7%) |
| Slow switching | 33 (33.7%) | 22 (42.3%) | 9 (33.3%) | 10 (23.8%) | 17 (32.7%) | 6  (37.5%) | 2  (33.3%) |
| Did not get any specific advice | 12 (12.2%) | 5  (9.6%) | 8 (29.6%) | 3  (7.1%) | 7 (13.5%) | 1  (6.3%) | 0 |

Supplemental Table 1.
